# Statistics of Hospital and Out-Patient Service

**Published:** 1889-10

**Authors:** 


### STATISTICS OF HOSPITAL AND OUT-PATIENT SERVICE.

*Veterinary Department—University of Pennsylvania.*

September 1st, 1888, to September 1st, 1889.

| | HOSPITAL. | OUT PATIENTS. |
|---|---|---|
| Abscess | 7 | 5 |
| Adenitis | 1 | |
| Amputation | 1 | |
| Anæmia | 1 | 1 |
| Arthritis | 1 | 1 |
| Ascarides | 1 | 3 |
| Asthma | 2 | |
| Atrophy muscles | | 1 |
| Bronchitis | 2 | 10 |
| " chronic | 1 | |
| Castration | 11 | 6 |
| Cataract | 1 | 4 |
| Catarrh, auricular | 5 | 8 |
| Cerebral congestion | 1 | |
| Cerebro spinal meningitis | 2 | |
| Chorea | 3 | 4 |
| Collection of sinuses | 1 | 2 |
| " " gutteral pouches | 1 | |
| Conjunctivitis | 2 | 1 |
| Constipation | 10 | 3 |
| Contraction of flexor tendon | 1 | |
| Contraction of heels | 4 | 8 |
| Contusions | | 2 |
| Convulsions | 2 | 1 |
| Corns | 5 | 10 |
| " suppurating | 2 | |
| Curb | 2 | 2 |
| Cysts, serous | 1 | 5 |
| " mucous | 4 | 6 |
| Cystitis | | 1 |
| Deafness | | 1 |
| Debility | 2 | 1 |
| Diarrhœa | 2 | 1 |
| Dislocation of humera radial | 1 | |
| Distemper, dogs | 11 | 7 |
| Dropsy cerebral | | 1 |
| " abdominal | 2 | 4 |
| Dystocia | | 6 |
| Ears cut | 2 | |
| Eczema | 5 | 43 |
| Elephantiasis | | 4 |
| Emphysema | 1 | 6 |
| Enteritis, chronic | 4 | 1 |
| Epilepsy | 1 | |
| Exostosis fetlock | | 1 |
| Eye, external dislocation | 3 | 2 |
| Eye removed | | 1 |
| Feet deformed | 1 | 5 |
| Fistula of neck | | 3 |
| " " withers | 9 | 37 |
| " " clavicle | 2 | |
| Foreign body in throat, | 1 | 2 |
| " " " rectum | 1 | 1 |
| Fracture of femur | 6 | 1 |
| " " humerus | 2 | 6 |
| " " jaw | 2 | |
| " " maxilla | | 2 |
| " " nasal bones, | 1 | |
| " " metatarsus, | 1 | 1 |
| " " pelvis | 1 | 1 |
| " " radius | 1 | 2 |
| " " sesamoid bones | 1 | 1 |
| " " tibia | 9 | 21 |
| " " ulna | 1 | 1 |
| Gastritis | 8 | 9 |
| Gastro enteritis | 1 | 11 |
| Glanders | 5 | 8 |
| Goitre | 2 | 8 |
| Hæmatocele | 1 | |
| Hæmoglobinuria | 10 | |

| | HOSPITAL. | OUT PATIENTS. |
|---|---|---|
| Hernia inguinal | 1 | |
| Immobility | 2 | 5 |
| Impaction of rectum | 3 | 4 |
| Indigestion, intestinal | 4 | 10 |
| Influenza | 2 | 2 |
| Interfering | 2 | |
| Iritis plastic | 1 | |
| Keratitis | 5 | 26 |
| Keratoma | | 1 |
| Laminitis, acute | 2 | 12 |
| Laryngitis | | 2 |
| Locomotor ataxia | | 2 |
| Lymphadenoma | | 4 |
| Mange, sarcoptic | 23 | 19 |
| " follicular | 2 | |
| Meningitis | 3 | |
| Metritis | | 1 |
| Nail, street | 11 | 20 |
| Navicular disease | 5 | 23 |
| Neurotomy | 3 | |
| Necrosis | 1 | 2 |
| Opthalmia, periodic | | 10 |
| " traumatic | | 7 |
| Osteo porosis | 8 | 4 |
| Panopthalmitis | | 2 |
| Paralysis | 1 | |
| " penis | 1 | |
| Paraplegia | 1 | 1 |
| Penis, deformed | | 1 |
| Pharyngitis | 2 | 2 |
| Pleurisy, chronic | 2 | 1 |
| Pneumonia | 10 | 5 |
| " broncho | 3 | 17 |
| Poisoning, strychnia | 1 | 2 |
| Prolapsus rectum | 2 | |
| Poll Evil | 1 | 7 |
| Quarter cracks | | 19 |
| Quittor | 5 | 21 |
| Rabies | 31 | |
| " Suspicions of | 4 | |
| Rachitis | 1 | |
| Ringbone | 6 | 21 |
| Ring Worm | | 1 |
| Rheumatism | 4 | 11 |
| Roaring | | 6 |
| Schirrous cord | 4 | 4 |
| Shoeing | | 245 |
| Shoe boil | 1 | 5 |
| Side bone | 1 | 27 |
| Soundness—Examination for | 67 | 79 |
| Spavin | 26 | 30 |
| Spayed | 8 | 7 |
| Splints | 3 | 14 |
| Strangles | 1 | 8 |
| Strain, suspensory | | 5 |

| | HOSPITAL. | OUT PATIENTS. |
|---|---|---|
| Synovitis | 1 | 2 |
| " carpal sheath, | | 2 |
| Toenail deformed | | 7 |
| Tail deformed | 3 | 1 |
| Tail amputation | 7 | 2 |
| Tetanus | 3 | |
| Tenotomy | | 1 |
| Teeth filed | 1 | 37 |
| " irregular | | 14 |
| " caries | | 1 |
| " removed | | 3 |
| Testicle enlarged | 1 | |
| Thrush | 4 | 6 |
| Toe crack | 2 | 17 |
| Tongue lacerated | 1 | |
| Tuberculosis | 2 | |
| Tumor hypertrophy | | 2 |
| " epithelioma | | 1 |
| " fibroma | 1 | 10 |
| " hæmatoma | | 1 |
| " lipoma | | 1 |
| " lymphoma | | 1 |
| " osteo sarcoma | | 1 |
| " papillometa | 2 | 3 |
| " sarcoma | | 4 |
| " sebaceous | 1 | |
| " withers | 1 | 1 |
| " neuroma | | 2 |
| Ulcers | 2 | 3 |
| Uterus prolapsus | 1 | |
| Wounds, contused | | 3 |
| " gun-shot | 1 | 3 |
| " incised | 1 | |
| " lacerated | 15 | 21 |
| " surgical | 1 | |
| " punctured | 6 | 1 |
| | 497 | *1129 |
| Total | | 1626 |

| SPECIES OF ANIMALS. | HOSPITAL. | OUT PATIENT. |
|---|---|---|
| Horses | 265 | 811 |
| Mules | 12 | 54 |
| Ass | 1 | |
| Cows | 2 | |
| Goats | 2 | 2 |
| Pigs | 7 | 1 |
| Dogs | 190 | 238 |
| Cats | 15 | 23 |
| Badger | 1 | |
| Monkey | 1 | |
| Canary | 1 | |
| Total | 497 | 1129 |
| Total | | 1625 |

*Through carelessness of an employe the records of several hundred additional out patients were lost.

Cases Offering Special Points of Interest.—*No. 1511—March 20th, 1889.*—Sorrel gelding, 9 years, light driving horse; had met with an accident the day before during a runaway and could only walk on three legs. Unloaded with difficulty from the ambulance, the animal held up the near hind leg from the ground. Inside of fetlock a cut about two and a half inches long ran obliquely over the lower border of the internal sesamoid bone through the skin and synovial sheath and severed the lateral sesamoid ligament, the perforatus tendon two-thirds of its width, and the perforans one third. A finger could be carried down the great sesamoid sheath and up between the sesamoid bones and the anterior face of the perforans tendon. A moderate discharge of synovia and blood oozed from the wound. The off heel on the outside had sustained a severe cut through the coronary band and into the lateral cartilage. General condition much disturbed. Not eating, depressed, coat dry, R. 18, P. 48, temp. 102° F. The wounds were washed clean, sprinkled with iodoform and bandaged with a good roll oakum.

*March 21st.*—Unable to put near foot to the ground, but eating, R. 16, P. 48, temp. 100½° F. The discharge has become thicker and of a rich yellow color. March 22d and the next day the animal remained in the same condition and received only local treatment, but on the 24th the discharge became much more profuse and the animal showed marked symptoms of pain and fever. Irrigation was applied to the wound and quiniæ sulph., iod. bicrab. administered internally. On the 25th the temperature was 105 1-5° F., the respirations 24 and the pulse 60. The fever and pain continued with slight variations for some days. During this time the discharge of synovial continued thick, almost jelly-like. The treatment consisted of diuretics and laxatives with quiniæ internally, irrigation through the day and dressings of iodoform and oakum at night. The discharge continued of the same character, but the opening gradually closed until it became a mere fistula through which the secretion came only when the animal was forced to put weight on the member. The general symptoms improved slowly until the day the opening closed about April 10th, when marked improvement took place. Considerable swelling existed all around the fetlock and pastern, but this rapidly disappeared with irrigation, painting with tr. iodine and firm bandaging. The animal was turned to pasture the first of May, and in July was performing his ordinary work.

*No. 1533. April 6, 1889.*—Bay gelding, 8 years, 16.2 hands, cart horse, was thrown from railroad track, cart demolished and owner killed. Standing in yard, sweating profusely and unable to move; very nervous; R. 24, P. 44, temp. 102° F. Outside of off thigh a lacerated wound, large enough to admit the hand, reaching to the third trochanter which is splintered into several pieces. No other injuries to be found. Removed to hospital in ambulance, well rubbed and offered a warm mash which he refused. Fragments of bone removed and irrigation applied after external warmth has been revived. The animal could scarcely be led to and from irrigation stall, being unable to lift the leg either forwards or backwards. Irrigation, with injections of carbolized oil, was continued. Attention paid to diet, with the use of salines for some days, during which the temperature ranged from 100½° to 103½° F. After a few days a space the size of the palm of one's hand could be felt on the external and posterior surface of the femur, completely denuded. In about two weeks the external wound had so closed that it became necessary to make a counter opening at a more dependant point, a few spiculæ of bone coming away and then the wound healed rapidly, leaving a deep, depressed cicatrix apparently attached to the bone, but the animal was entirely free from lameness even at a trot.

*No. 1548. April 9th, 1889.*—Bay gelding, 6 years, saddle horse, received a kick, from a sharply calked shoe, on near shoulder, making a large wound and fracturing the deltoid crest. This case followed the same course as the one above, but more rapidly, and after the removal of several pieces of bone the size of a hazelnut, the wound healed, and the horse was working in six weeks, not lame.

These two cases show the great *vis medicatrix naturæ* in horses, as compared with man, in bone injuries.

